# COVID-19 Conspiracies, Trust in Authorities, and Duty to Comply with Social Distancing Restrictions

**DOI:** 10.1007/s43576-021-00042-x

**Published:** 2022-01-11

**Authors:** Kristina Murphy, Molly McCarthy, Elise Sargeant, Harley Williamson

**Affiliations:** 1 Griffith Criminology Institute, Griffith University, Brisbane, Australia; 2 School of Social and Political Sciences, University of Melbourne, Melbourne, Australia

**Keywords:** COVID-19, Trust, Conspiracy theories, Duty to comply

## Abstract

In 2020 governments worldwide implemented various laws and social distancing restrictions to contain the spread of the COVID-19 virus. At the same time, conspiracy theories emerged purporting that authorities were using the COVID-19 pandemic to permanently control or harm citizens. These conspiracies undermined government responses to the pandemic and in some cases elicited civil disobedience. Using survey data from 779 Australians collected eight months into the pandemic, we examined the relationship between conspiracy beliefs, trust in the government, and duty to comply with authorities during the COVID-19 pandemic. We also examined whether trust in government moderated the association between conspiracy beliefs and duty to comply. We found that those prone to conspiracy theory beliefs and who distrusted government were less likely to comply with authorities during the pandemic. We also found that trust in the government moderated the negative relationship between conspiracy beliefs and duty to comply; high trust served as a protective factor against conspiracy beliefs. Importantly, we found that how government actions were experienced and perceived during the pandemic were important correlates of Australians’ level of trust in the government. Our findings point to the importance of governments maintaining high trust in their efficacy and approach during a crisis.

## Introduction

The first official case of COVID-19 was recorded in China in November 2019. In the ensuing months COVID-19 spread around the world and a global pandemic was declared. Governments moved to implement extraordinary laws and social distancing restrictions in a bid to limit the spread of the virus. In Australia, these measures included closing Australia’s international border, instituting mandatory hotel quarantine for returning Australians, limiting the movement of citizens across state borders, instituting periodic snap lockdowns, encouraging good hygiene (e.g. hand-washing, mask wearing), urging citizens to engage in social distancing (see Murphy et al., 2020; Mazerolle & Ransley, 2021), and more recently, encouraging citizens to vaccinate.

During this early period of the pandemic, uncertainty and misinformation surrounding the COVID-19 virus took hold. Conspiracy theories circulated through social media, with a common theme that governments were exaggerating the seriousness of the virus in order to permanently control or harm citizens (Imhoff & Lamberty, 2020). Conspiracy theories can undermine public trust in authorities, reduce public engagement in pro-social behaviours, and increase the incidence of antisocial behaviours (Ardevol-Abreu et al., 2020; Imhoff & Lamberty, 2020; Imhoff et al., 2021; Jolley & Douglas, 2014; Jolley et al., 2019). Indeed, anti-government protests, reduced adherence to social distancing regulations, and COVID-19 vaccine hesitancy or refusal have all been attributed to the uptake of conspiracy theory beliefs during the pandemic (e.g. Imhoff & Lamberty, 2020; Keane, 2020; Orzechowski et al., 2021; Pummerer et al., 2021; Romer & Jameison, 2020; Roozenbeek et al., 2020). The success of virus containment measures are contingent on people-trusting authorities and adhering to their advice.

Our study examines the relative importance of both conspiratorial thinking and trust in government on Australians’ duty to comply with authorities during the COVID-19 pandemic. It also tests whether trust in government moderates the association between conspiratorial thinking and duty to comply with virus containment measures. Assuming that trust in government is important, we identify factors associated with trust in the government during the first eight months of the pandemic. Before presenting our findings, the following section outlines what we know about conspiracy theories and their consequences. This is followed by a discussion on trust in authorities during a crisis and importantly why we expect trust to moderate the negative consequences of believing in conspiracy theories.

### Conspiracy Theories: Their Causes and Consequences

Conspiracy theories attempt to explain significant events and circumstances. They can be distinguished from other accounts of events in two important ways. First, they explain events by referencing the malevolent acts of powerful groups who manage to conceal their role (Douglas et al., 2017). Second, conspiracy theories tend to be sceptical of any ‘official’ accounts of events, no matter what the evidence suggests (Bartlett & Miller, 2010).

Psychological literature suggests that people are drawn to conspiracy theories when important *psychological* needs are not being met. Douglas (2021) suggests these needs might include the desire to satisfy curiosity or to reduce feelings of anxiety, powerlessness, or psychological uncertainty (see also Van Prooijen, 2018). Douglas (2021) also suggests that beliefs in conspiracy theories can restore a threatened sense of security and control or can be used to hold oneself or one’s group in positive regard. *Political* and *situational* factors are also important for understanding who might be drawn to conspiracy theories. Political conservatives are consistently more likely to believe in conspiracies than political liberals (Uscinski et al., 2020). People who feel more uncertain about the state of the world are also more prone to believe in conspiracy theories (Miller, 2020; Van Prooijen & Douglas, 2017). Yet, people from a range of backgrounds and in different situations believe in conspiracy theories. Hence, conspiracy theories are “not solely the domain of extremists and paranoids” (Miller, 2020, p.327).

Numerous studies explain the causes of conspiracy theory beliefs. Yet comparatively fewer studies examine the negative *behavioural consequences* of conspiratorial thinking. Of those that do, researchers show that conspiracy believers are less likely to comply with social norms (Imhoff & Lamberty, 2020). Imhoff et al. (2021) found that a conspiracy mindset made it more likely that people engaged in illegal, non-normative forms of political action (see also Imhoff & Bruder, 2014). Holding a conspiracy-prone worldview has also been linked to greater willingness to accept violence as a justifiable course of action and to engage in criminal behaviour (Jolley & Paterson, 2020; Jolley et al., 2019).

Miller (2020, p. 327) argues that the COVID-19 pandemic has presented “a perfect storm” for activating the psychological, political, and situational factors responsible for the development of conspiracy theories. Common COVID-19 conspiracies include the belief that governments have exaggerated the seriousness of the virus to permanently control or harm citizens; that Bill Gates has created a tracking device which is inserted into COVID-19 vaccines; that the virus was created as a biological weapon to reduce the world’s population; that 5G towers spread the virus; and that COVID-19 is a hoax (Imhoff & Lamberty, 2020). Recent studies show that stronger COVID-specific conspiracy beliefs are associated with a reduced propensity to engage in preventative health behaviours (e.g. Imhoff & Lamberty, 2020) or to vaccinate against the virus (e.g. Romer & Jameison, 2020; McCarthy et al., 2021b). Jolley and Paterson (2020) also found that believing 5G towers spread COVID-19 was linked to stronger willingness to vandalise 5G towers. With such negative consequences, this begs the question: Can anything be done to reduce the negative consequences of conspiratorial thinking? We propose that *trust* in authorities is critical to this goal.

### The Importance of Trust in Authorities During a Pandemic

Trust is essential for promoting peoples’ duty to support and obey authorities in a crisis (Volkan, 2014). This is because trust can assist in reducing perceived uncertainty and risk associated with that crisis (Colquitt et al., 2011; Lin et al., 2016). When trust in authorities is high, people feel more obligated to support authorities (e.g. Murphy et al., 2014). During previous pandemics, studies showed that public trust in government fostered citizens’ compliance with public health advice (e.g. Prati et al., 2011; Rubin et al., 2009). Rubin et al. (2009), for example, showed that people with higher trust in the UK government’s handling of the 2009 H1N1 influenza outbreak were more likely to alter their health behaviours (e.g. regularly washed hands, self-isolated if sick). More recently, Pagliaro et al. (2021) studied behavioural intentions during the COVID-19 pandemic across 23 European countries and found that trust in authorities was positively associated with peoples’ intentions to comply with prescribed COVID-19 prevention behaviours (e.g. self-isolating) and discretionary behaviours (e.g. giving money to charities to help tackle COVID-19).

Trust is therefore important in a pandemic, but it is fragile and can be damaged. Esaiasson et al. (2020) cited research on reactions to the 2009 swine flu epidemic (H1N1) in the Netherlands and found that institutional trust decreased significantly at the peak of the crisis (Van der Weerd et al., 2011). More recently, Davies et al. (2021) found that trust in the UK government initially rose in the early months of the COVID-19 pandemic but declined steadily after that. Healy and Malhotra (2009) note that citizens often find reasons to blame governments for their lack of efficacy or efficiency during a crisis, which could explain declining trust in governments over time during a pandemic.

Studies show that trust is heavily influenced by both *instrumental* and *normative* factors (Jackson & Bradford, 2009). Instrumental-based perspectives see trust linked to perceived competence and beliefs about the likelihood of receiving positive outcomes from authorities. During COVID-19, instrumental-trust concerns might be linked to judgements about how *effective* the government is in containing the spread of the virus or how well the police *deter* citizens from breaching health restrictions. This perspective suggests that citizens will trust authorities if they assume they or their loved ones will receive some future collective benefit from the government’s actions (e.g. not catching COVID-19).

Normative concerns also influence trust. Such concerns include the belief that authorities treat people respectfully and fairly, understand the needs of the community, and do not abuse or overstep legal authority. When citizens feel authorities are respectful and treat citizens fairly (i.e. *procedural justice*) they are more likely to trust those authorities (Murphy et al., 2014; Tyler & Huo, 2002). Procedural justice is important because it communicates symbolic messages about a person’s status in society (Tyler & Huo, 2002). Being treated disrespectfully or being subjected to heavy-handed enforcement signals to people that authorities view them as unworthy of fair treatment, which can elicit negative reactions (Tyler & Huo, 2002). Police officers—the visible arm of many governments’ COVID-19 enforcement response—have been criticised worldwide for using excessive force and abusive practices when enforcing COVID-19 restrictions (e.g. Daw, 2020; Mazerolle & Ransley, 2021). Such practices can damage trust in authorities.

People are also concerned with authorities recognising and respecting the limits of their power; what Trinkner et al. (2018) refer to as ‘*bounded-authority*’ concerns. Tyler and Trinkner (2017, p. 11) argue “that authorities’ directives can be rejected if they insist on trying to control behaviour outside appropriate domains”. Governments worldwide have adopted exceptional powers to enforce COVID-19 restrictions. Behaviours previously considered normal (e.g. socialising with friends/family; leisure travel) have been ‘criminalised’ during the pandemic, with authorities instituting fines for violations of COVID-19 restrictions. Such powers risk being seen as *heavy-handed* and might be viewed as overstepping the boundaries of normatively acceptable power. This could nurture fears of a turn to authoritarian rule, which can damage trust (Amat et al., 2020). Authorities therefore need to be cognizant of how their actions are perceived and experienced by citizens during a crisis, as they can promote or damage trust.

### How Might Conspiracy Beliefs and Trust be Related?

Studies suggest that individuals with low trust in government are more susceptible to develop conspiracy beliefs (van Prooijen et al., 2021). Low trust in government may signify vulnerability to developing conspiracy theory beliefs during the COVID-19 pandemic (Van Mulukom et al., 2020). At the same time, research also suggests that conspiracy theory beliefs undermine trust in authorities (Einstein & Glick, 2015). The causal relationship between trust in government and conspiratorial thinking has yet to be clearly established. Most likely, the relationship is reciprocal (van Prooijen et al., 2021).

Our aim is not to examine the causal relationship between conspiracy beliefs and trust in authorities. Rather, we seek to examine whether trust in authorities may protect against the negative influence of conspiracy theory beliefs on a person’s duty to comply with authorities during the COVID-19 pandemic. Specifically, we test whether trust in government *moderates* the negative relationship between conspiracy beliefs and duty to comply with government restrictions during COVID-19. Whilst we are unaware of any empirical studies that directly test this relationship, there is some theorising and empirical evidence from criminology that implies such an interaction should exist.

Research shows that trust in authorities becomes particularly salient when people feel uncertain, disengaged, or marginalised (i.e. the conditions present in conspiracy believers). For example, Murphy et al. (2009) demonstrated that when people question the legitimacy of an authority’s laws, they are less compliant with that authority. Murphy et al. also found that trust in the authority’s fairness moderated this relationship; the association between perceiving laws as illegitimate and non-compliance was weaker for those with high trust. In another study, Madon et al. (2017) found that individuals who felt more socially distant from police (disengaged) were more likely to report feeling obligated to obey police when they had high trust in police fairness; disengaged individuals with low trust in police were significantly less likely to report feeling obligated to obey police.

Social exchange theories posit that trust is vital for deepening relationships with authorities because it reduces uncertainty about the authority whilst fostering a sense of obligation (Blau, 1964). According to Colquitt et al. (2012, p.2), trust guides decisions about whether to cooperate and support authorities “when there is uncertainty about potential exploitation”. Colquitt et al. also note that high levels of trust can “mitigate or reduce the effects of virtually any form of uncertainty” (p.2), including concerns about the value of cooperating with an authority. From this perspective, trust may engender a stronger duty to comply and may instil a sense of comfort that reduces uncertainty more generally. Given that conspiracy beliefs often develop in response to psychological and situational uncertainty (Douglas, 2021), such theorising suggests that jurisdictions that can foster greater trust in governments during the COVID-19 pandemic might be able to reduce the negative consequences of conspiracy beliefs.

### The Current Study

**Fig. 1:**
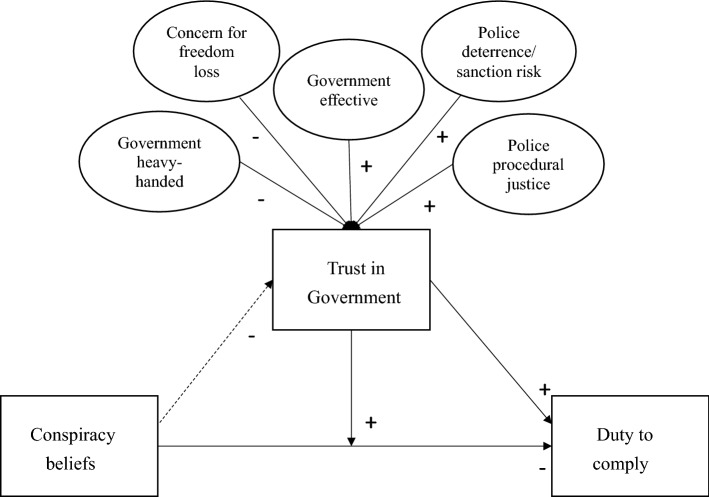
Conceptual framework tested

Our study highlights how governments can effectively bolster trust and duty to comply with authorities during a significant health crisis. Few conspiracy theory studies to date have examined the potential consequences that conspiratorial thinking patterns have on *illegal behaviours* (for exceptions see Jolley et al., 2019; Pummerer et al., 2021). By situating the current study within the COVID-19 pandemic—where compliance with social distancing rules is expected by law—this study allows an exploration of how conspiracy beliefs are related to citizens’ duty comply with specific COVID-19 laws. It also examines for the first time how trust in government *interacts* with conspiracy beliefs to influence duty to comply with these laws. Hence, the current study will contribute to our understanding of how people respond to government actions and performance during a public health crisis and how this relates to their trust in government. Figure 1 presents the conceptual framework to be tested. We test three hypotheses:

#### Hypothesis 1

Conspiracy theory beliefs and distrust of government will both be associated with reduced duty to comply with authorities and their COVID-19 rules.

#### Hypothesis 2

Trust in government will serve as a protective factor, moderating the negative association between conspiracy theory beliefs and duty to comply.

#### Hypothesis 3

Both instrumental and normative concerns will be more strongly associated with enhanced trust in the government, than believing in conspiracy theories.

#### Study Context

**Fig. 2:**
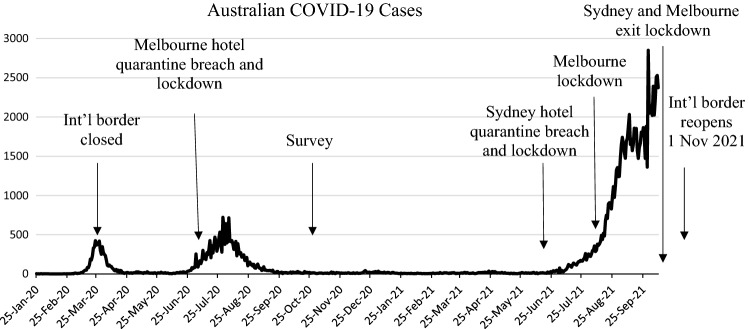
Timeline of Australia’s COVID-19 case numbers

Our study is situated in Australia; thus it is important to provide context around the COVID-19 restrictions leading up to the time of data collection, as well as what has happened since then. This study uses survey data collected in Australia between 22 October and 12 November 2020—eight months into the pandemic. Figure 2 presents where in Australia’s timeline of the pandemic the survey was fielded. Prior to data collection in October 2020 Australia had largely been successful in containing the spread of the COVID-19 virus (i.e. 27,495 confirmed cases and 905 deaths). This was likely due to the fact that Australia’s international border was closed on 20 March and the requirement of returning Australians to undertake 14-days of mandatory hotel quarantine. However, Melbourne residents had experienced an extended period of lockdown due to hotel quarantine breaches and rising cases (112 days), with lockdown restrictions only beginning to lift during the survey fielding period (see Fig. 2). Other states had only experienced brief lockdowns up until this time.

Subsequent to data collection, Australia’s success in containing the virus began to wane. Public complacency surrounding COVID-19 restrictions, a slow vaccine rollout, and repeated virus breaches from mandatory hotel quarantine led to two states (New South Wales and Victoria) experiencing high daily case numbers (i.e. 136,371 additional cases and 543 additional deaths between October 2020 and October 2021) and further extended lockdowns. Our findings therefore report on factors associated with trust and compliance *prior* to these major outbreaks.

## Methodology

### Participants and Procedure

Survey participants were recruited nationally through Facebook’s AdManager function and surveys were completed anonymously online by directing participants to a Limesurvey weblink. Only Australian Facebook users aged 18 + were eligible to participate. Of the 2,004 Facebook users who clicked on the Limesurvey link, only 1435 participants submitted their survey responses (71.6% response rate). However, after removing participants who had not completed the survey in full (*n* = 548) or who had incorrectly answered an ‘attention check’ question (*n* = 108), a final useable sample of 779 participants was achieved. This represents a 38.9% adjusted response rate.

Participants ranged in age from 18 to 83 (*M* = 54.3; *SD* = 12.5), 46.6% were men, 73.7% were born in Australia, and most were Caucasian (94.4%). Whilst the survey drew on a national convenience sample of Australian Facebook users, survey response demographics were compared with the most recent 2016 Australian census. Survey respondents were generally representative of the Australian population on gender and country of birth, but older and more educated respondents were over-represented (see Table 1). Survey respondents were from all states and territories in Australia; however, those living in Victoria were also over-represented. Perhaps this occurred because Melbourne residents had just experienced an extended lockdown prior to data collection and may have wanted to express their opinions about the Victorian government.

**Table 1:** Sample characteristics compared to Australian population characteristics

Variable	Sample %	Census %
Gender		
Female	52.9	50.7
Male	47.1	49.3
Age		
15–34^a^	7.3	33.4
35–54	38.8	32.8
55 +	53.8	31.3
Country of birth		
Overseas	26.3	26.3
Australia	73.7	73.7
Educational attainment		
No high school	5.9	24.5
High school	11.4	15.7
Trade certificate	13.7	24.7
University	48.5	22
State of Residence		
Northern Territory	0.6	1
New South Wales	12.6	32
ACT	1.2	1.7
Queensland	24.4	20
Victoria	47.2	25.7
South Australia	4	7
Western Australia	7.1	10.5
Tasmania	3	2.1

^a^Only those older than 18 were eligible to participate in our study

### Measures

The survey contained 201 questions, gauging public perceptions of restrictions introduced by the Australian Government to address the COVID-19 pandemic, participants’ general attitudes towards authorities (i.e. police, health authorities, federal, and state/territory governments) and their handling of the COVID-19 pandemic. The survey also included questions about conspiracy beliefs, the impact the virus had on participants’ lives, and a range of demographic background variables. All multi-item scales used in the current study were subjected to a factor analysis, with no cross-loading detected (see Table 2). A mean score was computed for all multi-item scales. Exact wording of measures used are presented below or in Table 2.

**Table 2:** Factor analysis of multi-item scale measures

	Factor			
Items	1	2	3	4
Duty to comply with authorities *To what extent do you think that it is everybody's duty to support the authorities (e.g. Government and police) during the COVID-19 pandemic by…*				
Abiding fully with all current COVID-19 restrictions	.73			
Ensuring you maintain 1.5-m physical distance from others when out and about	.93			
Wearing a mask when out if required to do so	.75			
Avoiding travel to a COVID-19 hotspot	.83			
Avoiding crowded places (e.g. shopping malls, sporting events, social gatherings, parties)	.96			
Staying at home as much as possible	.90			
Avoiding greeting people with a handshake, kiss, or hug	.91			
Not socialising with friends and family if not allowed to do so	.77			
Providing your contact details at cafes, restaurants, and bars	.81			
Trust in Government				
I have confidence in my State/Territory Government		.95		
I trust my State/Territory Government to act in the best interests of all Australians		.94		
I generally support the decisions made by my State/Territory Government		.90		
My State/Territory Government usually acts in ways that are consistent with my own ideas about what is right and wrong		.92		
How much confidence do you have in the ability of the following institutions to handle the COVID-19 pandemic? [My State/Territory Government]		.69		
Police procedural justice *When issuing fines to people flouting social distancing rules, I think police in my State/Territory have generally….*				
Treated people with dignity and respect			.92	
Displayed compassion and understanding			.95	
Made decisions based upon facts, not personal biases			.89	
Taken account of people’s explanations for why they are where they are before issuing a fine			.91	
Treated people fairly			.95	
Conspiracy mentality				
I think that many important things happen in the world, which the public is never informed about				.75
I think that politicians usually do not tell us the true motives for their decisions				.65
I think that government agencies closely monitor all citizens				.73
I think that there are secret organisations that greatly influence political decisions				.83
Eigenvalues	13.76	2.07	1.66	1.40
% of variance	59.83	9.00	7.21	6.08

Principal axis factoring with oblimin rotation; all factor scores > .30 displayed

### Duty to Comply with Authorities During COVID-19

The 9-item ‘duty to comply’ scale was a dependent variable in this study. It captured participants’ sense of duty and obligation to support authorities during the COVID-19 pandemic by complying with various social distancing restrictions. Respondents were asked the extent to which they agreed that it is everybody’s duty to support authorities (i.e. government and police) by *not* engaging in nine restricted behaviours during the pandemic (measured on a 1 = not at all everybody’s duty to 5 = completely everybody’s duty scale). Higher scores on this scale indicate a stronger duty to support authorities by complying with the restrictions (M = 3.27; SD = 1.50; alpha = 0.97).

### Trust in Government

Trust in government served as both a dependent and an independent variable and was measured using four items. Three items asked about participants’ general trust in their state/territory government (measured on a 1 = strongly disagree to 5 = strongly agree scale) and one item asked about confidence in their State/Territory government’s ability to handle the pandemic (measured on a 1 = not confident at all to 5 = a lot of confidence scale). Trust in the eight *state/territory* governments was measured (as opposed to the Australian Federal Government’s response to COVID-19) because each state/territory within Australia was responsible for overseeing its own virus containment measures and hotel quarantine and there was considerable variability in success between states/territories. Higher scores indicate greater trust in participants’ respective state/territory government (*M* = 2.36; SD = 1.40; alpha = 0.97).

### Conspiracy Mentality

Research suggests that individuals differ in their susceptibility to believe in conspiracy theories. This susceptibility is known as a conspiracy mentality, which is highly correlated with specific conspiracy theory beliefs (Bruder et al., 2013)*.* A four-item conspiracy mentality measure was adapted from the work of Bruder et al. (2013). Items were measured on a 1 = strongly disagree to 5 = strongly agree scale, with higher scores indicating enhanced belief in conspiracy theories (*M* = 3.89; SD = 0.93; alpha = 0.84).

### Belief in COVID-Specific Conspiracies

Nine COVID-specific conspiracy theories were presented (see Table 3) and participants were asked the extent to which they agreed with each conspiracy (measured on a 1 = strongly disagree to 5 = strongly agree scale). A higher score on each item suggests participants more strongly believed in the specific COVID-19 conspiracy (*M* = 2.72; SD = 1.26; alpha = 0.94).

### Instrumental-Trust Concerns

Survey respondents’ instrumental-trust concerns relevant to the COVID-19 pandemic included perceived sanction risk (i.e. deterrence) and perceived government effectiveness. These two concepts reflect perceived government action in response to the pandemic.

#### Sanction Risk

Police in each of the eight Australian states/territories were given powers to arrest and issue infringement notices to those caught flouting COVID-19 restrictions. Participants were asked: ‘How likely is it that someone would get caught and sanctioned by police for flouting COVID-19 restrictions?’; measured on a 1 = not at all likely to 5 = very likely scale. This item assessed the perceived ability of authorities to *deter* people from breaching COVID-19 restrictions. A higher score on this item indicates greater perceived sanction risk (M = 2.19; SD = 0.79).

#### Government Effectiveness

Respondents were asked one question about their own state/territory government’s effectiveness in containing the spread of COVID-19 (‘My State/Territory Government has been effective in containing the spread of COVID-19’); measured on a 1 = strongly disagree to 5 = strongly agree scale. A higher score indicates the participants’ respective government was perceived as more effective (*M* = 3.00; SD = 1.62).

### Normative-Trust Concerns

Respondents’ normative-trust concerns regarding how Australian authorities had dealt with the COVID-19 pandemic up to the time of data collection were measured via three constructs: concern about freedom loss, government heavy-handedness, and police procedural justice. These variables measure perceived government action and reflect concerns regarding perceived abuse of power and procedural justice.

#### Concern About Freedom Loss

This construct was measured via one item and assessed how worried respondents were about their freedoms being permanently curtailed by the government (‘How much do you worry that after the whole COVID-19 pandemic ends, our freedoms will never return to what they were before the COVID-19 outbreak?’; measured on a 1 = not worried at all to 5 = extremely worried). This measure reflects concerns about the government overstepping the boundaries of normatively acceptable power. Higher scores indicate greater concern for freedom loss (*M* = 3.59; SD = 1.60).

#### Government Heavy-Handed

Respondents were asked one question about whether they felt their state/territory government had been heavy-handed during the pandemic (‘My State/Territory Government has been heavy-handed in how they have dealt with the COVID-19 pandemic’; measured on a 1 = strongly disagree to 5 = strongly agree scale). Higher scores suggest respondents felt the government had been heavy-handed/abused their authority (*M* = 3.43; SD = 1.65).

#### Police Procedural Justice

Police were responsible for enforcing COVID-19 restrictions. Survey participants were presented with five questions asking about perceptions of police behaving in a procedurally just manner when issuing COVID-19 fines. Items were measured on a 1 = strongly disagree to 5 = strongly agree scale, with higher scores indicating police were perceived as more procedurally just (*M* = 2.68; SD = 1.29; alpha = 0.97).

### Demographic and Control Variables

Demographic and control variables were also included. Demographics included age, gender (0 = female; 1 = male), race (0 = other; 1 = Caucasian), country of birth (0 = overseas; 1 = Australia), educational attainment (1 = no/limited schooling to 9 = postgraduate degree), and state/territory of residence. A dummy variable was created for the state/territory of residence variable with Victoria set as the reference category (New South Wales, Queensland, Western Australia, Northern Territory, Australian Capital Territory, South Australia, and Tasmania were coded as ‘Other States/Territories’). Victoria served as the reference category because it endured the longest and strictest lockdown of all the Australian states/territories prior to fielding the survey. It was also the state with the highest number of COVID-19 cases (69%) and deaths (90%) at the time of data collection. For this reason, it is assumed that trust in government and support for authorities may have differed in this state compared to other states where case numbers were lower and where lockdowns and restrictions were relatively short-lived.

Control variables included political affiliation and job loss. Political affiliation was measured via one item: ‘Some people talk about “left” (e.g. Australian Labour Party, Greens), “right” (e.g. Liberal National Party, One Nation), and “centre” to describe political parties and politicians. With this in mind, where would you place yourself in terms of your support for political parties?’; measured on a 1 = very left-wing to 4 = centre to 7 = very right-wing scale (*M* = 4.14; SD = 1.49; 37.7% of respondents reported being left-leaning, 32.3% were centrist, and 39.9% were right-leaning). Job loss was measured as: ‘Have you lost your job/been stood down from your position due to COVID-19?’; measured as 0 = no, 1 = yes (13.2% (*n* = 103) responded ‘yes’).

## Results

### Descriptive Statistics

SPSS version 24 was used for all analyses. Table 3 presents the distribution of responses given for each of the nine COVID-19-specific conspiracy theories, which shows a high proportion of the sample believed in some COVID-19-specific conspiracies. The COVID-19 conspiracy receiving the highest endorsement was ‘Governments are using COVID-19 in a bid to permanently limit our freedoms’ (41.6% strongly agreed), whilst the conspiracy theory that received the least endorsement was ‘5G mobile networks increase our risk of catching COVID-19’ (only 2.2% strongly agreed).

**Table 3:** Proportion (%) of respondents who believe in COVID-19-specific conspiracy theories and bivariate correlation with conspiracy mentality scale

Conspiracy	Strongly disagree	Disagree	Neither disagree nor agree	Agree	Strongly agree	Correlation with conspiracy mentality scale*
The Government is exaggerating the seriousness of COVID-19	31.1	5.5	6.0	17.1	40.3	.62
Big pharmaceutical companies have exaggerated the seriousness of COVID-19 in a bid to make the rich richer	29.4	7.1	11.2	13.6	38.8	.66
Governments are using COVID-19 in a bit to permanently limit our freedoms	26.3	5.9	5.8	20.4	41.6	.69
COVID-19 was intentionally released by China as a biological weapon	30.4	8.7	26.8	16.7	17.3	.51
The COVID-19 death tally is being intentionally inflated	29.9	6.8	7.4	14.8	41.1	.64
COVID-19 is a scam dreamt up by global elites seeking to control the rest of us	46.7	8.0	15.5	13.0	16.8	.66
COVID-19 is NOT real	66.5	14.1	9.6	4.7	5.0	.46
5G mobile networks increase our risk of catching COVID-19	70.1	6.5	17.7	3.3	2.2	.47
COVID-19 vaccines will be used to harm or control society	44.9	7.2	13.0	13.5	21.4	.67

^*^all significant at *p* < 0.001

Table 3 also presents the bivariate correlations between the general ‘conspiracy mentality’ scale and each of the nine COVID-specific conspiracy beliefs. Prior research shows that a person’s susceptibility to believe in conspiracy theories (i.e. conspiracy mentality) is highly correlated with specific conspiracy theory beliefs (Uscinski & Parent, 2014). Our study confirms this. Table 3 shows that all nine COVID-specific conspiracies are positively correlated with the conspiracy mentality measure.^1^ A separate factor analysis (not reported here) revealed that the four conspiracy mentality items and the nine COVID-specific conspiracy measures formed one overall factor. Hence, to avoid multi-collinearity in subsequent regression analyses, only the general conspiracy mentality measure was used.

### Regressions

Two ordinary least squares (OLS) regression analyses were conducted. The first focused on Australians’ duty to support and comply with authorities and their COVID-19 restrictions during the pandemic. It addressed Hypotheses 1 and 2. The second regression focused on the instrumental and normative concerns (i.e. government actions) associated with Australians’ trust in government in the first eight months of the pandemic (addressing Hypothesis 3).

Prior to running these regressions, all scale variables were first screened for normality. No individual scale included in the ‘compliance’ or ‘trust’ regressions produced a skewness or kurtosis value greater than ± 0.70 or − 1.60, respectively (suggesting no problems with skewness or kurtosis). Assumptions of OLS regression including linearity, normality, and homoscedasticity were also assessed. Inspection of the scatterplots and bivariate correlations across the variables of interest showed the assumption of linearity was met. Calculation of the Durbin–Watson statistic indicated no issues with autocorrelation (Durbin–Watson = 2.05), the plots of the standardised residuals were within the normal range, and the plots revealed no major concerns for heteroscedasticity. As such, OLS regression was considered suitable for use with the data. Finally, multi-collinearity between independent variables was not detected in either regression model (i.e. no VIF score exceeded 1.70 in the ‘duty to comply’ regression or 2.30 in the ‘trust’ regression) and our study was sufficiently powered to detect both medium and large effects.

### Duty to Comply with Authorities During COVID-19

The first OLS regression focuses on Australians’ duty to support the government and police by complying with COVID-19 restrictions (see Table 4). Block 1 shows a number of demographic variables were associated with respondents’ felt duty to support the authorities. Older participants, those born in Australia, and those who had lost their jobs in the pandemic felt a greater duty to comply, whilst men and political conservatives felt less duty to comply. Victorians also expressed a stronger duty to comply than residents from other states/territories.

**Table 4:** OLS Regression with duty to comply with the authorities during COVID-19 as the dependent variable

	Block 1		Block 2	
Variable	b (se)	β	b (se)	β
*(Constant)*	2.592 (.310)***		2.775 (.306)***	
Age	.020 (.003)***	.168	.019 (.003)***	.158
Gender (ref: Female)	− .336 (.075)***	− .112	− .350 (.074)***	− .116
Race (ref: Caucasian)	− .105 (.162)	− .016	− .083 (.159)	− .012
Educational attainment	.032 (.022)	.037	.031 (.021)	.035
Country of birth (ref: Overseas born)	.209 (.083)*	.061	.197 (.082)*	.058
Political affiliation	− .160 (.028)***	− .158	− .165 (.027)***	− .163
No job loss	.282 (.109)**	.063	.270 (.107)*	.060
State of residence (ref: Victoria)	− .198 (.073)**	− .066	− .250 (.073)***	− .083
Trust in government	.443 (.034)***	.412	.470 (.033)***	.437
Conspiracy mentality/beliefs	− .409 (.048)***	− .252	− .469 (.048)***	− .288
Trust in government x Conspiracy mentality	−	−	.164 (.029)***	.145
*R*^2^	.569		.586	
Adjusted *R*^2^	.563		.580	
*R* change	.569		.017	
*F*	100.181***		31.753***	
df	760		759	

^*^p < 0.05; **p < 0.01; ***p < .001

Importantly, conspiracy beliefs and trust in government were both significantly associated with duty to comply. Those who scored higher on the conspiracy mentality measure expressed less duty to comply, whilst more trusting Australians were more likely to feel a duty to comply. Relative to all variables in the model, trust was most strongly associated with duty to comply.

**Fig. 3:**
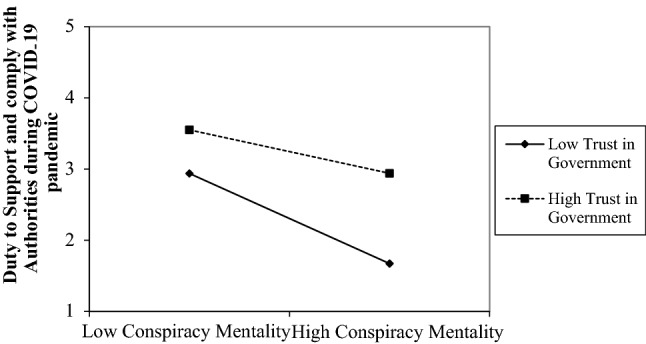
The relationship between conspiracy beliefs and duty to support the authorities during the COVID-19 as a function of trust in government

To explore whether trust *moderated* the negative association between conspiracy beliefs and duty to comply, an interaction term between conspiracy mentality x trust in government was entered in Block 2. It was positive and significant. Hence, simple slopes were computed at − 1 (low) and + 1 (high) standard deviations of the trust scale and are plotted in Fig. 3. Figure 3 shows that when participants scored high on the conspiracy mentality scale and also had low trust in government, they reported the least duty to comply with the authorities. Figure 3 also shows that high trust is associated with higher levels of duty to comply, irrespective of whether one scored low or high in conspiracy beliefs. Simple effects calculations revealed that the negative association between conspiracy beliefs and duty to comply was much weaker for those scoring *high* on trust (*b* = − 0.239 or *β* = − 0.147, *p* < 0.001; [95% CI − 0.348: − 0.129]) than for those scoring *low* on trust (*b* = − 0.699 or *β* = − 0.430, *p* < 0.001; [95% CI − 0.836: − 0.562]). This suggests that having high levels of trust in the government offers some protection against the negative consequences of believing in conspiracy theories on duty to comply with authorities and their COVID-19 restrictions.

### Trust in Government During COVID-19

A second OLS regression examined which instrumental and normative factors were most strongly associated with public trust in government during the first eight months of the COVID-19 pandemic (see Table 5). Demographic variables and the conspiracy mentality variable also served as predictors of *trust in government*.

**Table 5:** OLS Regression with trust in the government as the dependent variable

Variable	b (se)	β
*(Constant)*	2.312 (.340)***	
Age	− .002 (.002)	− .019
Gender (ref: Female)	− .042 (.056)	− .015
Race (ref: Caucasian)	− .105 (.120)	− .017
Educational attainment	− .025 (.016)	− .030
Country of birth (ref: Overseas born)	− .122 (.061)*	− .038
Political affiliation	− .057 (.021)**	− .061
No job loss	− .118 (.080)	− .028
State of residence (ref: Victoria)	− .309 (.058)***	− .110
Conspiracy mentality/beliefs	− .099 (.038)**	− .066
*Government action variables*		
Sanction risk (deterrence)	.073 (.025)**	.056
Government effectiveness	.385 (.024)***	.447
Concern for freedom loss	− .151 (.027)***	− .172
Government heavy-handedness	− .133 (.022)***	− .157
Police procedural justice	.170 (.031)***	.157
*R*^2^	.736	
Adjusted *R*^2^	.731	
*F*	150.830***	
df	756	

^*^p < 0.05; **p < 0.01; ***p < .001

Table 5 shows that political conservatives and those born in Australia were significantly less likely to trust the government during COVID-19 than political liberals or those born overseas. Relative to Victorians, residents in the ‘Other States/Territories’ were also more likely to trust the government, whilst those who scored high on the conspiracy mentality scale were less trusting of government. All five instrumental and normative concern variables were significantly associated with trust. If the government was seen as more effective in handling the COVID-19 pandemic (instrumental concern), when the risk of being sanctioned for violating COVID-19 restrictions was perceived as higher (deterrence; instrumental concern) and when police were perceived as more procedurally just during the pandemic (normative concern), respondents were more likely to trust the government. In contrast, when the government was perceived as being heavy-handed in their pandemic approach (normative concern) or when participants expressed concern about freedom loss post-pandemic (normative concern), they were less likely to trust the government. Together, these findings suggest that authorities’ actions during the pandemic are important for promoting and maintaining public trust in the government. Finally, an examination of the regression coefficients suggests that trust in government is more strongly associated with what authorities do and how they behave during the COVID-19 pandemic, whilst conspiratorial thinking appears to matter less.

## Discussion

Our study attempted to shed light on how governments can effectively bolster public trust and compliance during a crisis and when conspiracy theories are prevalent. We examined these relationships in the context of the COVID-19 pandemic in Australia. Specifically, we examined whether trust in government may moderate the relationship between conspiracy beliefs and Australians’ duty to comply with the authorities (government and police) during the COVID-19 pandemic. Our findings suggest trust is critically important in influencing Australians’ duty to comply with authorities and further that high trust in government may diminish the negative effect of conspiracy beliefs on duty to comply, confirming a moderation effect. Hence, our study also sought to identify the instrumental and normative concerns that shaped Australians’ trust in the government during the pandemic.

### Findings and Implications

As expected, we found that believing in conspiracy theories and greater distrust in the government were both associated with reduced duty to support and comply with the authorities during the COVID-19 pandemic, including with legally mandated restrictions (Hypothesis 1 supported). As such, our findings add to evidence suggesting that conspiracy theory beliefs and distrust of government institutions both reduce public compliance with laws (c.f., Murphy et al., 2014; Pummerer et al., 2021). In support of Hypothesis 2, we also found a significant interaction effect between conspiracy mentality and trust in government on duty to comply. Individuals with a *high* conspiracy mentality and who also had *high* levels of trust in the government felt a stronger duty to comply when compared to those who scored *high* on conspiracy mentality but *low* on trust. This finding suggests that trust in government is important for reducing the negative consequences of conspiratorial thinking on peoples’ duty to comply with authorities during a crisis.

As posited by social exchange theories, high levels of trust in authority guides citizens’ decisions about whether to cooperate and support authorities “when there is uncertainty about potential exploitation”. Colquitt et al. (2012) argue that because trust acts as an uncertainty reducer it promotes obligation to obey authorities through its ability to instil a sense of comfort, reducing concerns about an exploitative authority. Our findings support the importance of fostering government actions that bolster and maintain public trust, to counteract the negative impacts of conspiracy theories.

People place their trust in authorities and governments specifically to protect them from harm during a crisis (Perrin & Smolek, 2009). Yet Healy and Malhotra (2009) note that citizens often turn on governments as crises unfold. For example, Davies et al. (2021) found that trust in the UK government fell significantly during the COVID-19 pandemic. Our study highlights the types of concerns that either damaged or promoted Australians’ trust in their own state/territory government during the first eight months of the COVID-19 pandemic.

As expected, those with a strong conspiracy mentality were less likely to trust the government, aligning with recent findings in this area (Meuer & Imhoff, 2021; Pummerer et al., 2021). More importantly, however, perceived government actions and performance were more consequential for encouraging public trust, providing support for Hypothesis 3. Specifically, believing the government had been effective in managing COVID-19, believing credible sanctions were in place to deter people from flouting COVID-19 restrictions, and perceiving the police—the enforcement arm of the government—as more procedurally just when enforcing the governments’ COVID-19 restrictions were each positively associated with trust in government eight months into the pandemic in Australia. Conversely, being concerned that the government was using the pandemic to permanently limit Australians’ freedoms and believing that the government had been heavy-handed in their response to the pandemic were both negatively associated with trust. The findings suggest that governments might actively bolster trust and potentially diminish the effect of conspiracy theory beliefs, if they focus on (a) communicating the *efficacy* of their public health responses, (b) ensuring that police enforcement embodies *procedural justice* principles, and (c) considering *public concerns about civil liberty violations and over-reach*.

Research suggests that conditions and events that make individuals feel powerless or uncertain can give rise to conspiracy beliefs (Douglas, 2021; Van Prooijen, 2018; Van Prooijen & Jostmann, 2013). Hence, trustworthy actions by governments that can engender a sense of empowerment and certainty may be able to reduce such beliefs. Van Prooijen (2018) specifically theorised about the importance of procedurally just responses from authorities for increasing a sense of empowerment and the perceived fairness and morality of authorities under conditions of uncertainty. He proposed such responses may reduce the negative emotional states of powerlessness and uncertainty that appear to precondition the uptake of conspiracy theory beliefs. Our findings provide empirical evidence supporting Van Prooijen’s argument and offer practical recommendations for authorities as they continue to navigate through the COVID-19 pandemic as well as for future pandemics.^2^

In response to addressing public concerns about the government violating citizens’ civil liberties (i.e. *freedom loss*), our research suggests that clear communication about the timeframes and conditions under which COVID-19 restrictions will be rescinded is one way to address public concerns that authorities are intentionally overstepping the boundaries of normatively acceptable power in a bid to permanently control citizens (see Van Der Bles et al., 2019). Limiting perceptions of the *heavy-handedness* of government actions may be harder to achieve, however. The public health response in Australia and particularly the use of numerous restrictive lockdowns has been perceived as heavy-handed by some (including many of our survey participants) (c.f., Han et al., 2020). Whilst lockdowns can take a heavy toll on people’s mental health and the economy, such measures have been effective in reducing community transmission of COVID-19 in several Australian outbreaks (for evidence of this see Mannix, 2021). It would be unwise, therefore, to recommend that authorities avoid using lockdowns altogether. But again, clear communication about 1) why lockdowns and restrictions are necessary to protect the community, 2) when these lockdowns and restrictions will be rescinded (and following through on this as soon as practicable), and 3) how effective they have been in stemming the spread of the virus might all prove helpful in allaying citizens’ concerns about heavy-handedness.

In sum, our findings suggest that *how* authorities utilise their regulatory powers during a crisis is critical to engendering and maintaining public trust and compliance with public health directives (McCarthy et al., 2021a; Murphy et al., 2020). Indeed, a large body of research speaks to the role of the perceived fairness and effectiveness of authorities and their use of power, in enhancing public trust (e.g. Murphy et al., 2014; Tyler & Huo, 2002; Tyler & Trinkner, 2017). The findings of our study suggest that these factors remain important for generating public trust in the context of the COVID-19 pandemic. Moreover, greater trust in the authorities (government and police) appears to provide protection against the negative consequences of believing in conspiracy theories.

Finally, our findings highlight some key differences between Victorian residents and Australians living in other states/territories. We found that Victorians felt a greater duty to support authorities but also expressed greater distrust in the government, relative to residents in other states/territories. To understand these findings, it is important to situate them within the context of the COVID-19 pandemic in Australia. Despite an initial Australia-wide hard lockdown from March to May 2020, along with closures to state and international borders and a range of other social distancing measures, most Australian states and territories did not endure long periods of lockdown in 2020. However, a number of virus leaks from quarantine hotels for returning Australians resulted in notable community transmission in Victoria. This led Victorians into a second full lockdown in July 2020 (Mercer, 2020). Victorians remained in the second lockdown for 112 days, with restrictions finally easing in October 2020 as virus cases fell (just prior to our survey data collection).

The fact that Victorians expressed a greater duty to support and comply with the authorities in the first eight months of the pandemic may have been a function of the more serious health threat that Victorians faced during this period, alongside the strong government messaging focused on the need for collective action to limit the spread of COVID-19. Yet a substantial proportion of Victorians had their trust in government eroded by negative perceptions of government effectiveness (e.g. incompetent management of quarantine hotels), perceived police enforcement unfairness, and perceived over-reach of authority. Once again, our results speak to the importance that the perceived effectiveness, fairness, and appropriateness of authorities’ power and actions play in securing public trust in the government.

When considering our findings as a whole, it is important to note that Australians are generally very trusting of government. This was also the case during the COVID-19 pandemic (see Goldfinch et al., 2021). Whether our findings would be replicated in low-trust countries is unclear. Our findings imply that in countries where trust in government is low, conspiracy theories may take hold of citizens and negatively impact their compliance with COVID-19 restrictions. If this is so, how might authorities in low-trust countries respond? Bicchieri et al. (2021) may have an answer. In a study of nine countries, they revealed that in low-trust countries, citizens’ trust in government was weakly associated with their self-reported compliance with COVID-19 restrictions, but trust in scientists was strongly associated with compliance. This suggests that in low-trust countries, government authorities may have better success in implementing unpopular COVID-19 restrictions if they foster citizens’ trust in scientists and be upfront in how their pandemic decisions and policies have been heavily guided by science. But whether trust in scientists would overcome the negative consequences of conspiratorial thinking in such countries still remains to be empirically validated.

### Limitations

Before concluding, our study has some limitations that should be considered. First, our sample was recruited *online* via Facebook. Australia has poorer internet coverage in rural and regional areas (Nirmalathas, 2016), which may have limited our ability to attract respondents living outside major cities. Further, whilst the sample was still somewhat representative of the general Australian population on key demographics, Facebook has been a magnet for those spreading COVID-19 conspiracies (Bruns et al., 2020; Evershed et al., 2021). It is possible that, compared to an alternative recruitment strategy, our study attracted more people prone to conspiratorial thinking. We would argue, however, that for a study focused on the consequences of conspiratorial thinking this recruitment methodology was perhaps advantageous. Facebook offered an efficient means for recruiting a sufficient sample holding firm conspiracy theory beliefs (see Table 3), overcoming criticisms that previous conspiracy theory studies typically under-represent conspiracy theorists (see Douglas & Sutton, 2018). But the views expressed by our respondents may not be reflective of the broader Australian community, thus reducing the generalisability of our findings. Future research should replicate our findings using a representative population sample. Second, our survey data were cross-sectional in nature. Caution needs to be taken to understand the temporal relationships between our variables. For example, whether conspiracy thinking causes reduced duty to comply with authorities or vice versa cannot be ascertained with our data. The associations in our study could be further explored through a longitudinal cohort study. Third, the survey was conducted eight months into the pandemic and after Australia’s second-wave outbreak. The results may have been different if the survey was conducted later in the pandemic and after experiencing more severe outbreaks and repeated lockdowns.

### Conclusion

Despite some limitations, the findings add to our understanding of the consequences of conspiracy theory beliefs on compliance with legal mandates. They also provide insight into government responses that may mitigate the impact of such beliefs. Our study suggests that maintaining high levels of public trust in government might protect against the negative consequences of believing in conspiracy theories on duty to comply with authorities and their laws in a public health crisis. We argue that trust is important because it can reduce feelings of uncertainty experienced by those who condone conspiracy beliefs. Our findings also suggest that trust in authorities may not be an entirely fixed or enduring trait for conspiracy theorists. Rather, the perceived effectiveness, fairness, and appropriateness of government actions seem able to counter the negative effects of conspiracy theory beliefs on people’s trust in authorities.

## Footnotes

1 An additional analysis (not reported here) revealed no discernible differences in belief in COVID-19 conspiracy theories for residents in Victoria versus the other states/territories of Australia.

2 Perceptions of police procedural justice during the pandemic could have been influenced by the recent Black Lives Matter movement. Australians held a number of protests during the COVID-19 pandemic to condemn police brutality toward people of colour, particularly First Nations citizens. The movement could have made issues of police bias (i.e. procedural injustice) more salient to the average Australian, thereby affecting their procedural justice evaluations of pandemic policing in the current study.
